# Carotid Atherosclerosis in Chronic Kidney Disease: Pathophysiological Mechanisms, Prevention and Tailored Clinical Management

**DOI:** 10.3390/jcm15103909

**Published:** 2026-05-19

**Authors:** Iulia Nastase, Traian Iordachi, Maria-Magdalena Gurzun, Cristian Gutu, Nicolae Sarbu

**Affiliations:** 1 Faculty of Medicine and Pharmacy, “Dunărea de Jos” University of Galați, 800008 Galați, Romania; 2 The School for Doctoral Studies in Biomedical Sciences, “Dunărea de Jos” University of Galați, 800008 Galați, Romania; 3 Department of Cardiology, University of Medicine and Pharmacy Carol Davila, 8 Eroii Sanitari Boulevard, 050474 Bucharest, Romania; 4 “Dr. Aristide Serfioti” Emergency Military Hospital, 800150 Galați, Romania; 5 “Sf. Ioan” Emergency Clinical Pediatric Hospital, 800487 Galați, Romania

**Keywords:** chronic kidney disease, carotid atherosclerosis, primary prevention, cardiovascular risk, stroke prevention, blood pressure control, lipid-lowering therapy, SGLT2 inhibitors, lipoprotein(a), vascular calcification, arterial stiffness, endothelial dysfunction, screening strategies

## Abstract

The cardiovascular risk imposed by chronic kidney disease is significantly enhanced, and carotid atherosclerosis is an early indicator of systemic vascular damage. In this review, we summarize available data relative to primary prevention strategies for carotid atherosclerosis in chronic kidney disease (CKD) with a focus on risk-adapted and stage-specific management. We conducted a narrative review of the literature. A structured literature search was performed in major databases (PubMed, Scopus, Web of Science and Google Scholar), focusing on studies published between 2012 and 2025, including observational studies, randomized controlled trials, and international guideline recommendations. The review focuses on blood pressure management, lipid-lowering therapy, glycemic control, antiplatelet therapy, as well as lifestyle interventions and screening strategies in patients with CKD without a history of cerebrovascular events. CKD-specific processes, such as inflammation, endothelial dysfunction and vascular calcification, may influence the progression of carotid plaques, highlighting the need to improve traditional and non-traditional risk factor management. The focus of prevention continues to emphasize blood pressure (BP) and lipid control as well. At the same time, routine carotid screening and systematically implemented antiplatelet therapy have no known benefit, but the potential for elevated bleeding risk, especially in advanced CKD. Primary prevention should therefore focus on optimal medical treatment, as well as disease-specific strategies according to CKD stage. Additional CKD-specific studies with carotid endpoints are necessary.

## 1. Introduction

Chronic kidney disease is recognized as a major modifier of cardiovascular risk, extending beyond traditional risk factors and acting as a potent cardiovascular risk enhancer [1,2,3,4]. Patients with CKD face a much greater burden of atherosclerotic cardiovascular disease, with cardiovascular events remaining the main cause of morbidity and mortality, no matter the stage of renal dysfunction [1,5,6]. This excess risk is not entirely explained by conventional risk factors, suggesting the contribution of CKD-specific pathophysiological mechanisms that accelerate vascular damage [2,3,7,8,9].

Carotid atherosclerosis is a readily accessible and clinically relevant manifestation of systemic atherosclerosis [8,10,11,12]. Structural changes in the carotid arteries, including increased intima–media thickness and the presence of atherosclerotic plaques, have been associated with an elevated risk of ischemic stroke and other adverse cardiovascular outcomes [8,12,13]. As such, carotid atherosclerosis serves not only as a local vascular pathology but also as a surrogate marker of generalized atherosclerotic disease burden [10,13,14,15,16]. In patients with CKD, carotid atherosclerosis appears to develop earlier and progress more quickly, reflecting the combined effects of traditional cardiovascular risk factors and non-traditional CKD-related contributors such as chronic inflammation, endothelial dysfunction, and vascular calcification [2,4,10,13,14,15,16,17,18].

Despite the recognized association between CKD and accelerated atherosclerosis, evidence specifically addressing carotid atherosclerosis in this population remains limited [12,15,19,20]. Strategies in preventing carotid atherosclerosis are extrapolated from studies conducted in the general population, in which patients with moderate-to-advanced CKD are often underrepresented or excluded [10,15,21,22,23]. Furthermore, available studies frequently focus on secondary prevention or composite cardiovascular outcomes, rather than on the primary prevention of carotid atherosclerotic disease as a distinct entity [21,22,24,25,26,27]. This gap in the literature complicates clinical decision-making and underscores the need for tailored management in patients with carotid atherosclerosis and CKD [5,10,15].

Compared with the general population, carotid atherosclerosis in CKD patients tends to occur earlier, progress more rapidly, and reflects a higher burden of both traditional and CKD-specific cardiovascular risk factors, thereby potentially representing a more aggressive phenotype of systemic vascular disease [2,4,10,24,25]. This review focuses exclusively on primary prevention strategies for carotid atherosclerosis in CKD patients without prior cerebrovascular events. For consistency, the term “carotid atherosclerosis” is used throughout the manuscript as the primary descriptor of carotid vascular disease.

## 2. Materials and Methods

### 2.1. Research Strategy

This review was conducted using a structured narrative approach to ensure transparency while maintaining the narrative (non-systematic) nature of the review. A comprehensive literature search was performed using major international databases, including PubMed, Scopus, Web of Science and Google Scholar. The search focused on randomized controlled trials, prospective and retrospective cohort studies, case–control studies, systematic reviews, meta-analyses, and international clinical practice guidelines addressing carotid atherosclerosis and cardiovascular prevention in patients with chronic kidney disease (CKD).

Priority was given to studies published in English between 2012 and 2025, with particular emphasis on evidence from the last 5–7 years. Earlier landmark studies were included when considered essential for understanding the pathophysiological mechanisms or therapeutic foundations relevant to carotid atherosclerosis in CKD. Search terms included combinations of the following keywords: “chronic kidney disease”, “CKD”, “carotid atherosclerosis”, “carotid plaque”, “intima–media thickness”, “primary prevention”, “blood pressure control”, “lipid-lowering therapy”, “statins”, “SGLT2 inhibitors (sodium–glucose cotransporter 2 inhibitors)”, “antiplatelet therapy”, and “cardiovascular risk”. Reference lists of selected articles and relevant international guidelines (including KDIGO and ESC recommendations) were manually screened to identify additional pertinent studies. This manuscript represents a non-systematic narrative review, and no formal quality assessment or risk-of-bias evaluation of the included studies was performed. During the preparation of this manuscript, the authors used ChatGPT (OpenAI, GPT-5.3) for figure conceptualization.

### 2.2. Inclusion and Exclusion Criteria

Studies were selected based on predefined inclusion and exclusion criteria.

Inclusion criteria comprised:

- Studies evaluating carotid intima–media thickness, carotid plaque presence, plaque burden, plaque progression, or carotid-related cerebrovascular outcomes in patients with CKD;
- Studies assessing cardiovascular risk factors or preventive strategies relevant to primary prevention in CKD populations;
- Randomized controlled trials and high-quality observational studies reporting cardiovascular outcomes in CKD;
- International clinical practice guidelines addressing blood pressure, lipid management, glycemic control or antiplatelet therapy in CKD.

Exclusion criteria included:

- Studies focused exclusively on secondary prevention following established cerebrovascular events;
- Non-peer-reviewed publications, conference abstracts without full datasets;
- Studies lacking clear outcome definitions or sufficient methodological detail;
- Publications not available in English.

### 2.3. Study Selection

The initial database search yielded a broad range of records. After removal of duplicates, titles and abstracts were screened to exclude studies not directly related to carotid atherosclerosis or primary prevention in CKD. Although emphasis was placed on contemporary evidence, selected earlier landmark studies were retained to provide essential pathophysiological context and to support the interpretation of current preventive strategies. Particular attention was given to studies reporting carotid-specific endpoints (intima–media thickness, plaque presence, plaque thickness, or plaque progression) and their association with CKD stage and cardiovascular outcomes. The final selection reflects a synthesis of observational data, randomized clinical trials relevant to cardiovascular prevention in CKD, and current international guideline recommendations. Study selection and screening were performed by the authors, with final inclusion based on relevance to the topic and consistency with the objectives of the review. Approximately 50 relevant studies and international guideline documents were included in the final synthesis. No formal quality assessment or risk-of-bias evaluation was conducted, in line with the non-systematic narrative design of this review. Studies included in Table 1 were selected based on their relevance to carotid-specific endpoints, representation across different CKD stages, and overall clinical significance, with the aim of providing a concise overview of observational and longitudinal evidence in this field.

## 3. Pathophysiology of Carotid Atherosclerosis in Chronic Kidney Disease

### 3.1. Chronic Inflammation and Oxidative Stress

Chronic kidney disease is characterized by a persistent low-grade inflammatory state, which plays a central role in the initiation and progression of atherosclerosis [2,27,28,29,30]. Elevated circulating levels of inflammatory mediators, including C-reactive protein, interleukins, and tumor necrosis factor-α, contribute to endothelial activation and promote leukocyte adhesion within the vascular wall [29]. Also, increased oxidative stress resulting from impaired antioxidant defenses and enhanced production of reactive oxygen species accelerates lipid oxidation and foam cell formation [30]. Inflammation combined with oxidative stress creates a pro-atherogenic environment that facilitates early plaque development and progression in the carotid arteries of patients with CKD [24,25,30,31,32].

### 3.2. Endothelial Dysfunction and Arterial Stiffness

Endothelial dysfunction is a hallmark of CKD and represents a critical link between renal impairment and vascular disease [10,32]. The reduced bioavailability of nitric oxide, impaired vasodilatory capacity, and increased expression of adhesion molecules contribute to vascular inflammation and intimal thickening [11]. These alterations are accompanied by increased arterial stiffness, driven by structural changes in the vascular wall and medial calcification [32,33]. In the carotid circulation, arterial stiffening enhances pulsatile stress and shear forces, thereby accelerating atherosclerotic plaque formation and destabilization [18,31].

### 3.3. Disturbances in Mineral Metabolism and Vascular Calcification

Disorders of mineral metabolism are distinctive features of CKD and have substantial effects on the vasculature [8,33]. Hyperphosphatemia, secondary hyperparathyroidism, and alterations in fibroblast growth factor 23 and vitamin D metabolism promote the osteogenic differentiation of vascular smooth muscle cells [33]. Secondary hyperparathyroidism further contributes to vascular calcification through dysregulation of calcium–phosphate metabolism and stimulation of osteogenic transformation in vascular smooth muscle cells, thereby accelerating arterial stiffening and influencing cardiovascular risk [8,33,34]. This process leads to vascular calcification, a highly prevalent complication in CKD that contributes to arterial stiffening and also increases atherosclerotic plaque burden [33,34]. In the carotid arteries, calcific changes may coexist with lipid-rich plaques, further increasing cerebrovascular risk [18,22,35].

In addition to intimal atherosclerosis, medial arterial calcification (Mönckeberg calcinosis) represents a distinct and highly prevalent form of vascular disease in CKD, particularly in patients with diabetes. Unlike atherosclerosis, medial calcification primarily affects arterial stiffness rather than luminal narrowing but significantly contributes to cardiovascular risk [32,33,34].

### 3.4. CKD-Specific Dyslipidemia

Dyslipidemia in CKD differs qualitatively from that observed in the general population [35]. It is typically characterized by hypertriglyceridemia, reduced high-density lipoprotein cholesterol levels, and the predominance of small, dense low-density lipoprotein particles, which possess enhanced atherogenic potential [35]. In addition, impaired lipoprotein clearance and increased lipid oxidation further amplify vascular injury [35,36,37]. These alterations promote lipid accumulation within the arterial wall and contribute to the accelerated development of carotid atherosclerosis observed in patients with CKD, even in the absence of markedly elevated total cholesterol levels [24,25,35].

### 3.5. Lipoprotein(a) as a Non-Traditional Risk Factor in CKD

Lipoprotein(a) has gained recognition as a significant non-traditional cardiovascular risk factor with particular relevance in patients with chronic kidney disease [38]. Lp(a) levels are frequently elevated in CKD, reflecting reduced renal clearance and disturbances in lipoprotein metabolism, with concentrations tending to rise as kidney function deteriorates [38]. Beyond its atherogenic lipid content, Lp(a) exerts pro-inflammatory and pro-thrombotic effects through its apolipoprotein(a) component, which interferes with fibrinolysis and promotes vascular inflammation [38]. In the context of CKD, elevated Lp(a) levels have been associated with increased carotid intima–media thickness and a higher prevalence of carotid plaques, suggesting a contributory role in accelerated carotid atherosclerosis [20,24,38]. Standard lipid-lowering therapies, such as statins, have minimal impact on Lp(a) concentrations, highlighting a potential residual vascular risk [35]. Although specific therapeutic targets for lipoprotein(a) are not yet established in current clinical guidelines, elevated Lp(a) levels are recognized as a contributor to residual cardiovascular risk, particularly in high-risk populations such as patients with CKD [35,38].

## 4. Screening for Carotid Atherosclerosis in Primary Prevention

Screening for carotid atherosclerosis in asymptomatic individuals remains a controversial topic, particularly in populations considered to be at high cardiovascular risk [16,21,22]. Current evidence does not support routine screening for carotid artery disease in the general population, because the detection of asymptomatic carotid stenosis has not been shown to reduce the incidence of stroke or improve clinical outcomes when compared with optimized medical therapy alone [16,21,23]. Major preventive guidelines advise against the systematic use of carotid ultrasonography for primary prevention, even among high-risk groups [10,15,16].

Although chronic kidney disease is associated with accelerated atherosclerosis and an increased prevalence of carotid plaques [24,25], CKD alone does not justify routine carotid Doppler screening in asymptomatic patients [16,22]. The lack of randomized controlled trials demonstrating a clear benefit of screening in CKD limits the justification for widespread imaging in this population [21,24]. Furthermore, the identification of subclinical carotid disease may trigger additional diagnostic procedures or some interventions without proven benefit, potentially increasing patient risk, without clear outcome improvement [22,23].

Nevertheless, carotid imaging may have a role in selected clinical situations. Targeted screening can be considered for refined cardiovascular risk stratification in carefully selected patients, particularly when results are expected to influence the intensity of preventive medical therapy. This reflects the evolving nature of imaging-based risk assessment in CKD [20,22]. In addition, carotid ultrasonography remains a valuable tool in research settings, where it serves as a surrogate marker for systemic atherosclerosis and a measurable endpoint in interventional studies [19,20]. While current evidence and guideline recommendations do not support routine carotid screening in asymptomatic CKD patients, ongoing debate persists regarding its potential role in selected high-risk individuals. Emerging imaging approaches and improved risk stratification tools may further refine the clinical utility of carotid assessment in this population. However, at present, routine screening cannot be broadly recommended [16,21,22]. In the absence of robust evidence showing improved clinical outcomes, the theoretical advantages of routine carotid atherosclerosis screening in primary prevention are outweighed by the lack of demonstrated benefit. This supports a preventive strategy focused on comprehensive cardiovascular risk factor optimization rather than systematic imaging in asymptomatic patients [10,15,16].

## 5. Core Preventive Strategies

### 5.1. Blood Pressure Control

Blood pressure control represents the cornerstone of primary prevention of atherosclerotic cardiovascular disease in patients with chronic kidney disease [2,6,10]. Hypertension is highly prevalent in CKD and directly contributes to endothelial dysfunction, increased arterial stiffness, and accelerated atherosclerotic plaque formation [10,11]. The 2021 Kidney Disease: Improving Global Outcomes (KDIGO) guidelines recommend targeting a systolic blood pressure below 120 mmHg in adults with CKD [6]. Achieving optimal blood pressure control has been associated with reductions in arterial stiffness and may contribute to attenuating vascular remodeling and atherosclerotic progression by reducing pulsatile stress and shear forces within the carotid arteries [31].

However, intensive blood pressure reduction should be individualized, particularly in patients with advanced CKD, autonomic dysfunction, or frailty, in whom the risks of hypotension, compromised organ perfusion, and treatment-related adverse events may outweigh potential vascular benefits [9].

### 5.2. Lipid-Lowering Therapy

Lipid-lowering therapy may be a fundamental component of primary prevention strategies in CKD [10,12,35]. In non-dialysis CKD, statins, alone or in combination with ezetimibe, have demonstrated consistent reductions in major cardiovascular events, primarily based on evidence derived from CKD-specific populations as well as subgroup and post hoc analyses of large randomized trials [12,35]. According to current ESC/EAS guidelines, patients with CKD are generally classified as high or very high cardiovascular risk, with recommended LDL-C targets of <70 mg/dL or <55 mg/dL, respectively, depending on overall risk profile [12]. Chronic kidney disease is directly incorporated into cardiovascular risk stratification, with moderate CKD (eGFR 30–59 mL/min/1.73 m^2^) defining a high-risk category and severe CKD (eGFR < 30 mL/min/1.73 m^2^) a very high-risk category, thereby guiding the intensity of lipid-lowering strategies [12]. Beyond low-density lipoprotein cholesterol reduction, statins also exert pleiotropic effects, including anti-inflammatory actions and plaque stabilization, which may be relevant to atherosclerotic plaque stability and progression. However, carotid-specific outcomes are rarely reported, and much of the evidence supporting their use in this context is extrapolated from broader cardiovascular studies [35].

In contrast, the initiation of statin therapy in patients receiving maintenance dialysis has been associated with more modest or neutral cardiovascular benefits [29,39]. As a result, lipid-lowering treatment in this population should be considered on an individual basis, taking into account overall cardiovascular risk, life expectancy, and patient preferences [10,40,41].

### 5.3. Glycemic Control and Novel Therapies

Optimal glycemic control remains an important target in patients with diabetes and CKD [7,11]. Recent evidence has shifted attention toward glucose-lowering agents with proven cardiovascular and renal benefits [41,42]. Sodium–glucose cotransporter 2 inhibitors have been shown to reduce cardiovascular events in patients with CKD, largely independent of their glucose-lowering effects, based on evidence derived from CKD populations as well as post hoc analyses of cardiovascular outcome trials [41,42]. These benefits are thought to be mediated through hemodynamic, metabolic, and anti-inflammatory mechanisms, which may indirectly influence vascular remodeling and atherosclerotic progression. However, direct evidence linking these therapies to carotid-specific endpoints remains limited, and their potential effects on carotid atherosclerosis are inferred from general cardiovascular outcomes [30].

Non-steroidal mineralocorticoid receptor antagonists such as finerenone have demonstrated cardiovascular risk reduction in patients with diabetic CKD, likely through anti-inflammatory and anti-fibrotic pathways [43,44]. While direct evidence linking these agents to carotid plaque regression is limited, their favorable cardiovascular profile supports their role in comprehensive primary prevention strategies [11].

### 5.4. Antiplatelet Therapy

The use of antiplatelet therapy for primary prevention in patients with chronic kidney disease (CKD) remains controversial [16,21]. Current evidence does not support the routine use of aspirin in individuals without prior cardiovascular or cerebrovascular events, given the modest potential benefit and the consistently increased risk of bleeding [16]. This concern is particularly relevant in CKD, where platelet dysfunction, uremia-related alterations in hemostasis, and concomitant comorbidities further amplify hemorrhagic risk [28].

Importantly, in the context of primary prevention of carotid atherosclerosis, antiplatelet therapy should not be routinely prescribed in asymptomatic CKD patients, even in the presence of subclinical carotid plaques. Antiplatelet agents may be considered only in selected clinical scenarios that extend beyond pure primary prevention, such as patients with symptomatic carotid stenosis, where management falls under secondary prevention, or, in carefully selected cases, such as severe asymptomatic carotid stenosis, but only after individualized assessment of thrombotic and bleeding risk [10,21].

It should be emphasized that this review does not address secondary prevention strategies, including the management of symptomatic carotid stenosis or antiplatelet therapy following cerebrovascular events, but is strictly focused on primary prevention in CKD patients without prior cerebrovascular disease. Accordingly, for the majority of CKD patients without prior cerebrovascular events, primary prevention strategies should prioritize optimal medical therapy and comprehensive risk factor modification rather than routine antiplatelet use [10,16].

### 5.5. Lifestyle and Non-Pharmacological Interventions

Lifestyle modification constitutes an essential, though often underemphasized, component of primary prevention in CKD [10,35]. Dietary sodium restriction contributes to improved blood pressure control and may indirectly reduce vascular stiffness and atherosclerotic progression [35]. In addition, adherence to a heart-healthy dietary pattern, including reduced intake of saturated fats and balanced nutritional composition, may contribute to improved cardiovascular risk profiles and potentially influence the progression of atherosclerotic disease in patients with CKD [10,35]. Regular physical activity, adapted to the patient’s functional capacity and stage of kidney disease, has favorable effects on metabolic health, endothelial function, and overall cardiovascular risk [35]. Smoking cessation is very important, as tobacco exposure accelerates atherosclerosis and synergistically increases cerebrovascular risk in patients with CKD [10]. Collectively, these non-pharmacological measures reinforce the benefits of medical therapy and should be systematically integrated into preventive care [10].

## 6. CKD Stage-Specific Considerations

The heterogeneity of chronic kidney disease necessitates a stage-specific approach to the primary prevention of carotid atherosclerosis [5,10,35]. Cardiovascular risk, tolerance to preventive therapies, and competing comorbidities vary substantially across CKD stages, underscoring the importance of individualized management strategies [2,10]. A uniform preventive approach may lead to overtreatment in advanced disease or insufficient risk reduction in earlier stages [5,9].

In patients with early-stage CKD (stages 1–2), the emphasis should be placed on aggressive modification of traditional cardiovascular risk factors, including strict blood pressure control, lipid-lowering therapy when indicated, and comprehensive lifestyle interventions [6,12,35]. At this stage, preventive measures are generally well tolerated, and long-term cardiovascular benefits are most likely to be realized [1,10].

For individuals with moderate CKD (stage 3), statin therapy and optimized blood pressure control remain central components of primary prevention [6,12,35]. This stage represents a critical window for intervention, as cardiovascular risk accelerates while therapeutic responsiveness is still preserved [2,24]. Early identification and management of metabolic abnormalities and emerging risk factors may further attenuate the progression of carotid atherosclerosis [30,33]. In advanced CKD (stages 4–5), preventive strategies must be carefully individualized [6]. The balance between potential cardiovascular benefit and the risks associated with intensive therapy becomes increasingly narrow [9]. Avoidance of overtreatment is essential, particularly in frail patients or those with limited life expectancy, where aggressive risk factor modification may not translate into meaningful clinical benefit [5,10].

Among patients receiving maintenance dialysis, cardiovascular risk remains exceptionally high [2,29]; however, evidence supporting the initiation of certain preventive therapies, particularly lipid-lowering agents, is limited [29]. In this population, the primary focus should be on optimal blood pressure management, lifestyle optimization, and selective use of statins based on individualized risk assessment and patient preferences [5,6,35].

## 7. Discussion

**Figure 1:**
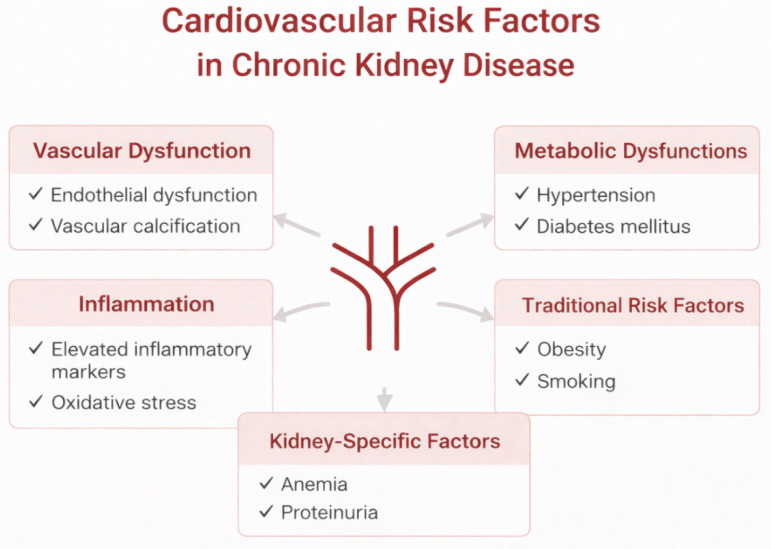
Pathophysiological mechanisms linking chronic kidney disease to carotid atherosclerosis (created by the authors).

Chronic kidney disease (CKD) is increasingly recognized as a major accelerator of systemic atherosclerosis, with carotid involvement representing a clinically accessible marker of cumulative vascular injury [1,2,18]. The pathophysiological substrate underlying carotid atherosclerosis in CKD extends beyond traditional cardiovascular risk factors and includes CKD-specific mechanisms such as chronic inflammation, oxidative stress, endothelial dysfunction, and disturbances in mineral metabolism [2,10,30]. These interrelated pathways promote early plaque development, vascular calcification, and increased arterial stiffness, ultimately amplifying cerebrovascular risk even in the absence of overt clinical events. Carotid atherosclerosis is closely associated with atherosclerotic involvement in other vascular territories, particularly coronary artery disease, reflecting the systemic nature of atherosclerosis. Several studies have demonstrated that carotid plaque burden correlates with coronary artery calcification and cardiovascular events, supporting its role as a surrogate marker of global atherosclerotic risk [45,46,47,48,49,50]. It should be acknowledged that much of the available evidence is extrapolated from general cardiovascular studies rather than derived from carotid-specific endpoints. Figure 1 illustrates the principal pathophysiological mechanisms linking CKD to accelerated carotid atherosclerosis. It highlights the complex interplay between CKD-specific and traditional cardiovascular risk factors, emphasizing the multifactorial nature of vascular injury in this population and the need for integrated rather than single-target preventive strategies.

Although kidney-specific factors such as proteinuria and anemia are recognized contributors to cardiovascular risk in CKD, their association with carotid atherosclerosis is primarily inferred from indirect evidence, as studies specifically evaluating carotid endpoints in relation to these factors remain limited.

**Figure 2:**
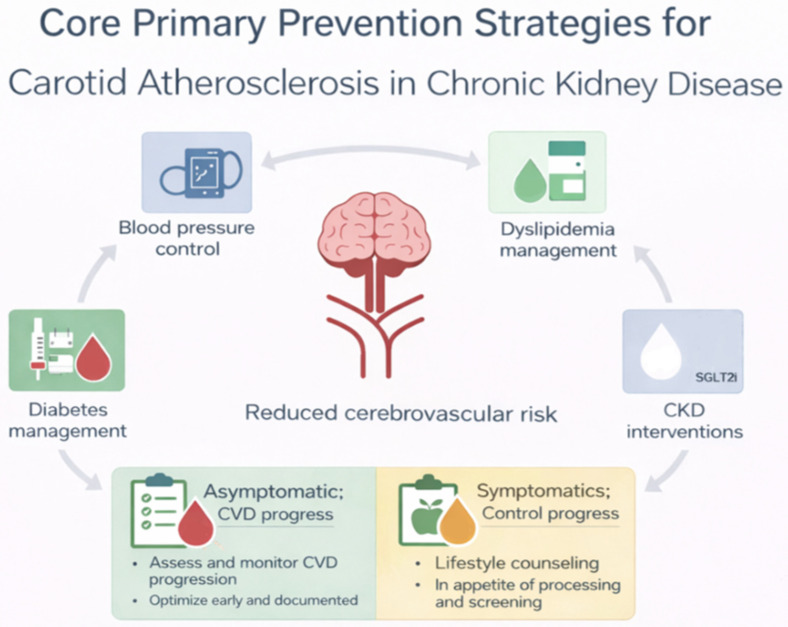
Core primary prevention strategies for carotid atherosclerosis in chronic kidney disease (created by the authors). SGLT2i: sodium–glucose cotransporter 2 inhibitors. CVD: cardiovascular disease. CKD: chronic kidney disease. The different colors of the raindrop-shaped graphics represent distinct categories of preventive strategies.

Although carotid ultrasonography can identify subclinical plaque burden, current evidence does not support routine screening for carotid atherosclerosis in asymptomatic CKD patients [16,21,22]. Randomized controlled trials demonstrating improved stroke outcomes with systematic carotid imaging are lacking, and guideline recommendations consistently discourage population-level screening strategies [10,15,16]. Instead, the available data support a prevention paradigm centered on global cardiovascular risk modification rather than lesion-directed screening approaches. Carotid atherosclerosis in CKD should therefore be interpreted primarily as a marker of systemic vascular vulnerability rather than an isolated therapeutic target. Figure 2 summarizes the core preventive strategies currently supported by available evidence. It highlights the central role of comprehensive risk factor modification, illustrating how multiple therapeutic domains converge to reduce overall cardiovascular risk rather than targeting carotid disease in isolation.

Blood pressure control remains the cornerstone of primary prevention [6,10]. In CKD, these previously described vascular alterations, including increased arterial stiffness and altered hemodynamic load, intensify pulsatile stress within the carotid circulation, thereby accelerating plaque development and progression [30,31,45,46]. Intensive systolic blood pressure reduction, when clinically appropriate, may attenuate vascular remodeling and slow atherosclerotic progression. However, the therapeutic window becomes narrower in advanced CKD, where frailty, autonomic dysfunction, and competing comorbidities necessitate individualized treatment targets [9].

**Figure 3:**
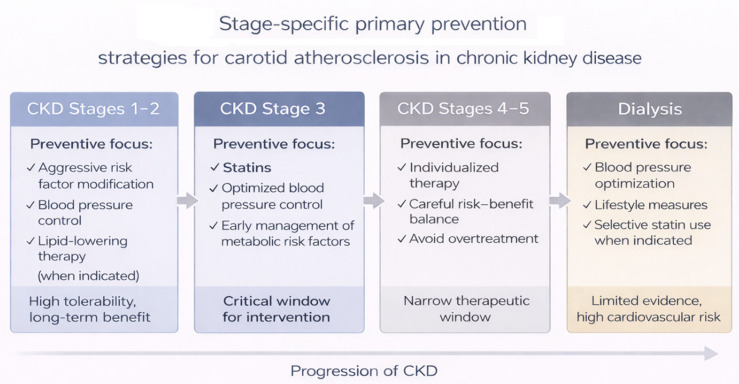
Stage-specific primary prevention strategies for carotid atherosclerosis in chronic kidney disease (created by the authors). CKD: chronic kidney disease. The arrows indicate the progression of chronic kidney disease and associated cardiovascular risk. The different colors represent distinct CKD stages and corresponding preventive strategies. Check marks highlight key therapeutic targets at each stage.

Lipid-lowering therapy represents another central component of preventive management. In patients with non-dialysis CKD, statins—with or without ezetimibe—have consistently demonstrated reductions in major cardiovascular events [12,35]. Although carotid-specific endpoints are rarely reported, the pleiotropic and plaque-stabilizing properties of statins provide mechanistic support for their role in limiting carotid plaque progression. In contrast, initiation of statins in dialysis patients has shown more modest or neutral cardiovascular benefits, underscoring the importance of stage-specific therapeutic decisions [29,35]. Figure 3 highlights the importance of adapting preventive strategies according to CKD stage. It reflects the dynamic balance between therapeutic benefit and potential risks across disease progression. However, the supporting evidence is not consistently derived from stage-stratified analyses, and these recommendations should therefore be interpreted primarily as conceptual guidance.

Beyond traditional cardiovascular risk factors, emerging cardio-renal protective therapies are reshaping the preventive landscape. Sodium–glucose cotransporter 2 (SGLT2) inhibitors and non-steroidal mineralocorticoid receptor antagonists have demonstrated significant cardiovascular risk reduction in CKD populations independent of glycemic control [41,42]. Although direct effects on carotid plaque morphology remain insufficiently characterized, their anti-inflammatory, hemodynamic, and anti-fibrotic properties—mechanisms previously discussed—suggest potential indirect benefits on vascular remodeling and atherosclerotic progression [30].

**Figure 4:**
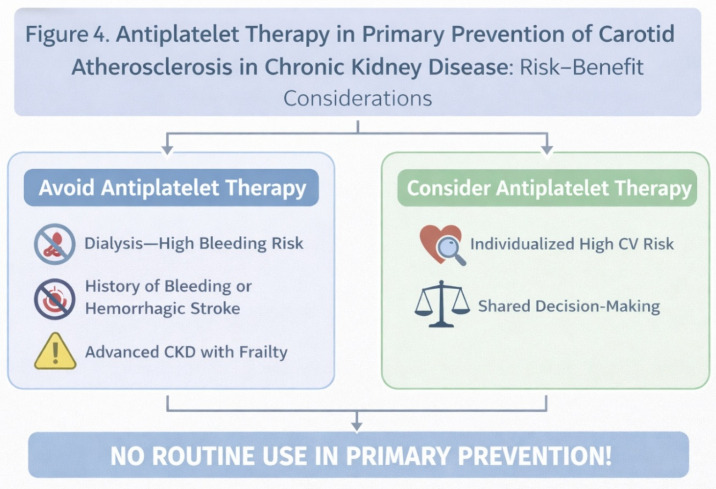
Antiplatelet Therapy in Primary Prevention of Carotid Atherosclerosis in Chronic Kidney Disease: Risk–Benefit Considerations (created by the authors).

The role of antiplatelet therapy in primary prevention remains particularly nuanced in CKD. Current evidence does not support routine aspirin use in CKD patients without established cardiovascular or cerebrovascular disease, given the modest potential benefit and consistently increased bleeding risk [16]. CKD-related platelet dysfunction and alterations in hemostasis further complicate the balance between thrombotic and hemorrhagic risk [28]. Accordingly, antiplatelet therapy should not be routinely prescribed for the primary prevention of carotid atherosclerosis and may be considered only in carefully selected high-risk individuals following individualized clinical assessment [10,21,47,48]. Figure 4 illustrates the risk–benefit considerations of antiplatelet therapy, highlighting the delicate balance between thrombotic and bleeding risks in CKD and supporting a cautious, individualized approach in the primary prevention setting.

**Figure 5:**
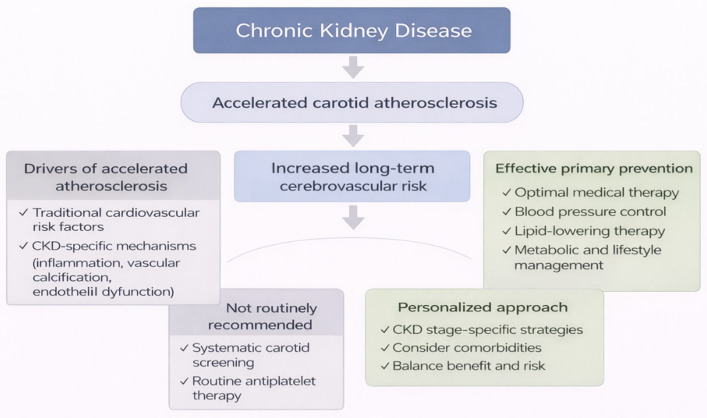
Key concepts in the primary prevention of carotid atherosclerosis in chronic kidney disease (created by the authors). The arrows indicate the progression from chronic kidney disease to accelerated carotid atherosclerosis and increased cerebrovascular risk. The different colors represent distinct components, including drivers of disease, clinical consequences, and preventive strategies. Check marks highlight key factors and therapeutic interventions.

The heterogeneity of CKD further complicates preventive strategies. Early-stage CKD allows for aggressive modification of cardiovascular risk factors with a favorable benefit–risk profile, whereas advanced CKD and dialysis require a more cautious therapeutic approach [5,10]. A uniform preventive strategy across all CKD stages risks undertreatment in early disease and overtreatment in advanced stages. Preventive interventions should therefore be individualized, integrating cardiovascular risk burden, life expectancy, and treatment tolerance [5,9]. Figure 5 summarizes the key conceptual principles underlying primary prevention of carotid atherosclerosis in CKD, highlighting the importance of a patient-centered and pathophysiology-driven approach to cardiovascular risk reduction in this population.

### Translating Evidence into Clinical Practice: The CEASE Framework

Given the multifactorial nature of vascular injury in CKD, primary prevention strategies must extend beyond isolated risk-factor management toward an integrated and stage-adapted approach. The evidence summarized in this review highlights the need to simultaneously address traditional cardiovascular risk factors, CKD-specific vascular mechanisms, and the considerable heterogeneity of cardiovascular risk across CKD stages.

**Figure 6:**
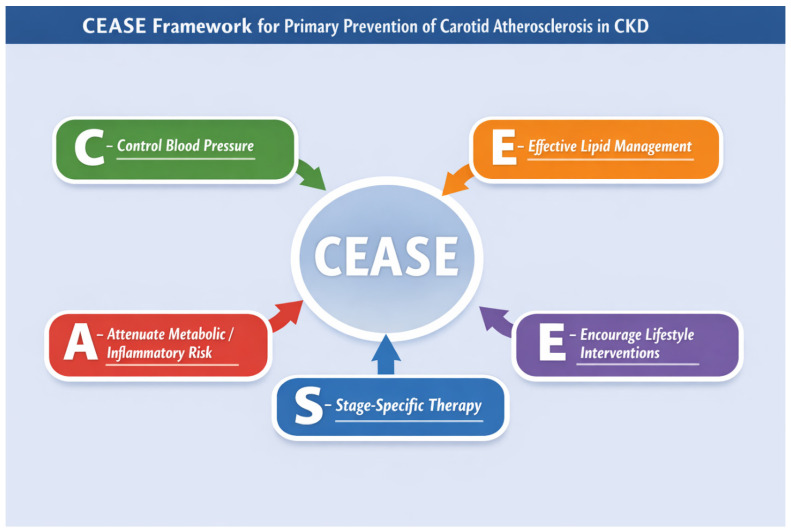
CEASE Strategy: Integrated and Stage-Specific Management of Carotid Atherosclerosis Risk in Chronic Kidney Disease (created by the authors). The arrows indicate the relationship between key preventive strategies and the central concept. The different colors represent distinct components of the framework. Uppercase letters correspond to the CEASE acronym: C–Control Blood Pressure; E–Effective Lipid Management; A–Attenuate Metabolic/Inflammatory Risk; S–Stage-Specific Therapy; E–Encourage Lifestyle Interventions.

To facilitate the translation of these principles into clinical practice, we propose the CEASE framework as a practical conceptual model for the primary prevention of carotid atherosclerosis in CKD (Figure 6). This framework encompasses five key domains: control of blood pressure, effective lipid management, attenuation of metabolic and inflammatory risk, stage-specific therapeutic adaptation, and encouragement of sustained lifestyle interventions.

Rather than representing a rigid therapeutic algorithm, CEASE should be viewed as a flexible, patient-centered framework that aligns pathophysiological mechanisms with targeted preventive strategies across the spectrum of CKD severity. By linking mechanistic insights with practical clinical actions, this approach may help clinicians translate current evidence into structured cardiovascular risk reduction strategies for patients with chronic kidney disease. The CEASE framework represents a conceptual model intended to facilitate the translation of current evidence into clinical practice and has not been prospectively validated.

Several limitations of the available evidence should be acknowledged. First, there is a lack of studies specifically designed to assess carotid-related endpoints, with most data derived from broader cardiovascular outcomes rather than direct measures of carotid atherosclerosis progression or regression. Second, substantial heterogeneity exists across CKD stages, with differences in cardiovascular risk profiles, treatment response, and competing comorbidities, which may limit the generalizability of preventive strategies. Finally, much of the current evidence is extrapolated from studies conducted in the general population or based on post hoc analyses, underscoring the need for CKD-specific trials with dedicated carotid endpoints.

While preventive strategies in CKD share similarities with those used in coronary artery disease, important distinctions exist. In contrast to coronary atherosclerosis, where imaging and interventional strategies are well established, carotid atherosclerosis in CKD remains primarily a surrogate marker rather than a direct therapeutic target. This further supports a prevention paradigm centered on global cardiovascular risk reduction rather than lesion-specific intervention.

## 8. Gaps in Evidence and Future Directions

### Limitations of Carotid-Specific Evidence in CKD

Despite increasing recognition of carotid atherosclerosis as a marker of systemic vascular disease in chronic kidney disease, direct evidence linking preventive strategies to carotid-specific outcomes remains limited. Most available studies primarily report composite cardiovascular endpoints, with carotid imaging parameters such as intima–media thickness or plaque characteristics often included as secondary or exploratory outcomes. Consequently, a substantial proportion of the preventive strategies discussed in this review are supported by evidence derived from broader cardiovascular studies rather than trials specifically designed to assess carotid atherosclerosis progression or regression. This distinction is important when interpreting the potential impact of therapeutic interventions on carotid vascular remodeling in patients with CKD. Therefore, current recommendations regarding the prevention of carotid atherosclerosis in CKD should be understood within the context of indirect evidence, highlighting the need for dedicated studies with carotid-specific endpoints.

Despite growing recognition of the high cardiovascular burden associated with chronic kidney disease, substantial gaps remain in the evidence base guiding the primary prevention of carotid atherosclerosis in this population [2,10,24]. One of the most significant limitations is the lack of randomized controlled trials specifically designed to evaluate carotid atherosclerosis-related endpoints, such as plaque progression, regression, or carotid-specific cerebrovascular outcomes [21,22]. Most available data are derived from studies focusing on composite cardiovascular endpoints or extrapolated from the general population, limiting their applicability to CKD-specific vascular pathology [10,15,24].

Patients with advanced CKD, including those with severe renal impairment or receiving dialysis, are consistently underrepresented in cardiovascular prevention trials [5,29]. This underrepresentation restricts the generalizability of existing evidence and complicates clinical decision-making in those at highest risk for both atherosclerotic events and treatment-related adverse effects [5,9]. As a result, preventive strategies in advanced CKD often rely on indirect evidence, observational data, or expert consensus rather than robust randomized data [5,10].

Future research should prioritize the identification and validation of biomarkers capable of improving cardiovascular risk stratification in CKD [30,38]. Novel circulating markers, including inflammatory mediators, mineral metabolism-related factors, and lipoprotein subfractions such as lipoprotein(a), may help identify patients at increased risk of carotid atherosclerosis progression [30,33,38]. In parallel, advances in vascular imaging techniques, including refined carotid ultrasonography protocols and multimodal imaging approaches, hold promise for enhancing the prediction of cerebrovascular risk beyond traditional clinical parameters [19,20].

Collectively, addressing these gaps through CKD-focused randomized trials, improved representation of advanced disease stages, and the integration of biomarker-driven and imaging-based risk assessment strategies will be essential to advance personalized primary prevention of carotid atherosclerosis in patients with chronic kidney disease [5,10,24]. Table 1 summarizes selected observational and longitudinal studies evaluating carotid atherosclerosis across the spectrum of chronic kidney disease, highlighting the heterogeneity of carotid endpoints and the consistent association between declining kidney function and increased atherosclerotic burden [47,48,49,50,51].

**Table 1:** Selected observational and longitudinal studies evaluating carotid atherosclerosis in chronic kidney disease.

Year	Study	Authors	Population	Carotid Endpoint	Main Findings
2012	Carotid plaque, carotid intima-media thickness, and coronary calcification equally discriminate prevalent cardiovascular disease in kidney disease [47].	Adeseun GA et al.	CKD (CRIC cohort)	cIMT, plaque, CAC	Demonstrated a significant burden of atherosclerosis among individuals with CKD.
2012	Carotid intima-media thickness in children with CKD: results from the CKiD study [48].	Brady TM et al.	Pediatric CKD	cIMT	cIMT was significantly higher in children with CKD vs. controls and independently associated with hypertension and dyslipidemia.
2019	Evaluation of carotid intima-media thickness and factors associated with cardiovascular disease in children and adolescents with chronic kidney disease [49].	Lopes R et al.	Pediatric CKD	cIMT	Increased cIMT prevalence was observed in children and adolescents with chronic kidney disease.
2021	Carotid plaque thickness is increased in chronic kidney disease and associated with carotid and coronary calcification [50].	Bjergfelt SS et al.	CKD stage 3	Plaque thickness	Carotid plaque thickness was higher in CKD stage 3 vs. controls and associated with CVD and vascular calcification.
2025	Progression of Carotid Intima-Media Thickness in Children of the Cardiovascular Comorbidity in Children With Chronic Kidney Disease Study: Risk Factors and Impact of Blood Pressure Dynamics [51].	Doyon A et al.	Pediatric CKD (4C)	cIMT progression	cIMT increased significantly over time and its progression was associated with blood pressure changes.

## 9. Conclusions

Chronic kidney disease is associated with accelerated development of carotid atherosclerosis, reflecting the combined effects of traditional cardiovascular risk factors and CKD-specific vascular mechanisms. Current evidence supports a primary prevention strategy centered on comprehensive cardiovascular risk factor control, particularly optimal blood pressure management, lipid-lowering therapy, and emerging cardio-renal protective treatments, rather than routine carotid screening in asymptomatic individuals.

Given the heterogeneity of CKD, preventive strategies should be tailored according to disease stage and patient-specific risk profiles. Future CKD-focused studies with carotid imaging or plaque-related endpoints are needed to refine risk stratification and guide targeted prevention strategies.

### Key Points

- Chronic kidney disease is a major accelerator of systemic atherosclerosis, with carotid involvement representing a clinically relevant marker of vascular risk.
- Direct evidence linking preventive strategies to carotid-specific outcomes in CKD remains limited, and most recommendations are extrapolated from general cardiovascular studies.
- Routine carotid screening is not recommended in asymptomatic CKD patients due to lack of demonstrated benefit.
- Primary prevention should focus on comprehensive cardiovascular risk factor control, particularly blood pressure management, lipid-lowering therapy, and emerging cardio-renal protective treatments.
- Preventive strategies should be individualized according to CKD stage, balancing potential benefits and risks.
- The CEASE framework provides a conceptual, stage-adapted approach to integrating preventive strategies in clinical practice.

## Data Availability

No new data were created or analyzed in this study. Data sharing is not applicable to this article.

## References

[1] GBD Chronic Kidney Disease Collaboration (2020). Global, regional, and national burden of chronic kidney disease, 1990–2017: A systematic analysis for the Global Burden of Disease Study 2017. Lancet.

[2] Jankowski J., Floege J., Fliser D., Böhm M., Marx N. (2021). Cardiovascular disease in chronic kidney disease. Circulation.

[3] Rangaswami J., Bhalla V., Blair J.E.A., Chang T.I., Costa S., Lentine K.L., Lerma E.V., Mezue K., Molitch M., Mullens W. (2019). Cardiorenal Syndrome: Classification, Pathophysiology, Diagnosis, and Treatment Strategies: A Scientific Statement From the American Heart Association. Circulation.

[4] Gansevoort R.T., Correa-Rotter R., Hemmelgarn B.R., Jafar T.H., Heerspink H.J.L., Mann J.F., Matsushita K., Wen C.P. (2013). Chronic kidney disease and cardiovascular risk: Epidemiology, mechanisms, and prevention. Lancet.

[5] (2024). Kidney Disease: Improving Global Outcomes (KDIGO) CKD Work Group. KDIGO 2024 Clinical Practice Guideline for the Evaluation and Management of Chronic Kidney Disease. Kidney Int..

[6] (2021). Kidney Disease: Improving Global Outcomes (KDIGO) Blood Pressure Work Group. KDIGO 2021 Clinical Practice Guideline for the Management of Blood Pressure in Chronic Kidney Disease. Kidney Int..

[7] (2022). Kidney Disease: Improving Global Outcomes (KDIGO) Diabetes Work Group. KDIGO 2022 Clinical Practice Guideline for Diabetes Management in Chronic Kidney Disease. Kidney Int..

[8] (2017). Kidney Disease: Improving Global Outcomes (KDIGO) CKD–MBD Update Work Group. KDIGO 2017 Clinical Practice Guideline Update for the Diagnosis, Evaluation, Prevention, and Treatment of Chronic Kidney Disease–Mineral and Bone Disorder (CKD-MBD). Kidney Int. Suppl..

[9] McEvoy J.W., Brouwers S., Bruno R.M., Brouwers S., Canavan M.D., Ceconi C., Christodorescu R.M., Daskalopoulou S.S., Ferro F.J., Gerdts E. (2024). 2024 ESC Guidelines for the management of arterial hypertension. Eur. Heart J..

[10] Visseren F.L.J., Mach F., Smulders Y.M., Carballo D., Koskinas K.C., Bäck M., Benetos A., Biffi A., Boavida J.-M., Capodanno D. (2021). 2021 ESC Guidelines on cardiovascular disease prevention in clinical practice. Eur. Heart J..

[11] Marx N., Federici M., Schütt K., Müller-Wieland D., Di Angelantonio E., Herrington W.G., Ajjan A.R., Kautzky-Willer A., Rocca B., Sattar N. (2023). 2023 ESC Guidelines for the management of cardiovascular disease in patients with diabetes. Eur. Heart J..

[12] Mach F., Baigent C., Catapano A.L., Koskinas K.C., Casula M., Badimon L., Chapman M.J., De Backer G.G., Delgado V., Ference A.B. (2020). 2019 ESC/EAS Guidelines for the management of dyslipidaemias: Lipid modification to reduce cardiovascular risk. Eur. Heart J..

[13] Aboyans V., Ricco J.-B., Bartelink M.-L.E.L., Björck M., Brodmann M., Cohnert T., Collet J.-P., Czerny M., De Carlo M., Debus S. (2018). 2017 ESC Guidelines on the Diagnosis and Treatment of Peripheral Arterial Diseases, in collaboration with the European Society for Vascular Surgery (ESVS). Eur. Heart J..

[14] Naylor A.R., Rantner B., Ancetti S., de Borst G.J., De Carlo M., Halliday A., Kakkos S.K., Markus H.S., McCabe D.J., Sillesen H. (2023). Editor’s Choice–European Society for Vascular Surgery (ESVS) 2023 Clinical Practice Guidelines on the Management of Atherosclerotic Carotid and Vertebral Artery Disease. Eur. J. Vasc. Endovasc. Surg..

[15] Bonati L.H., Kakkos S., Berkefeld J., de Borst G.J., Bulbulia R., Halliday A., van Herzeele I., Koncar I., McCabe D.J., Lal A. (2021). European Stroke Organisation guideline on endarterectomy and stenting for carotid artery stenosis. Eur. Stroke J..

[16] US Preventive Services Task Force (2021). Screening for Asymptomatic Carotid Artery Stenosis: US Preventive Services Task Force Recommendation Statement. JAMA.

[17] Bushnell C., Kernan W.N., Sharrief A.Z., Chaturvedi S., Cole J.W., Cornwell W.K., Cosby-Gaither C., Doyle S., Goldstein L.B., Lennon O. (2024). 2024 Guideline for the Primary Prevention of Stroke: A Guideline From the American Heart Association/American Stroke Association. Stroke.

[18] Gupta A., Baradaran H., Schweitzer A.D., Kamel H., Pandya A., Delgado D., Dunning A., Mushlin A.I., Sanelli P.C. (2013). Carotid plaque MRI and stroke risk: A systematic review and meta-analysis. Stroke.

[19] Saba L., Agarwal N., Cau R., Gerosa C., Sanfilippo R., Porcu M., Montisci R., Cerrone G., Qi Y., Balestrieri A. (2021). Review of imaging biomarkers for the vulnerable carotid plaque. J. Clin. Med..

[20] Johri A.M., Nambi V., Naqvi T.Z., Feinstein S.B., Kim E.S., Park M.M., Becher H., Sillesen H. (2020). Recommendations for the Assessment of Carotid Arterial Plaque by Ultrasound for the Characterization of Atherosclerosis and Evaluation of Cardiovascular Risk: From the American Society of Echocardiography. J. Am. Soc. Echocardiogr..

[21] Paraskevas K.I., Mikhailidis D.P., Antignani P.L., Baradaran H., Bokkers R.P., Cambria R.P., Dardik A., Davies A.H., Eckstein H.H., Faggioli G. (2022). Optimal management of asymptomatic carotid stenosis in 2021: The jury is still out. An international, multispecialty, expert review and position statement. Int. Angiol..

[22] Abbott A.L. (2022). Management of Patients with Asymptomatic Carotid Stenosis May Need to Be Individualized: A Multidisciplinary Call for Action. J. Stroke.

[23] Song P., Fang Z., Wang H., Cai Y., Rahimi K., Zhu Y., Fowkes F.G.R., Fowkes I.F.J., Rudan I. (2020). Global and regional prevalence, burden, and risk factors for carotid atherosclerosis: A systematic review, meta-analysis, and modelling study. Lancet Glob. Health.

[24] Bjergfelt S.S., Sørensen I.M.H., Urbak L., Kofoed K.F., Lange T., Feldt-Rasmussen B., Sillesen H., Christoffersen C., Bro S. (2024). Carotid plaque thickness predicts cardiovascular events and death in patients with chronic kidney disease. BMC Nephrol..

[25] Li W., Bai W., Miao C., Chen S., Zhang X., Fan Y., Li X., Wu S., Liu X., Hong J. (2022). Joint effects of carotid plaques and renal impairment on the risk of cardiovascular disease and all-cause death in a community-based population: A cohort study. Front. Cardiovasc. Med..

[26] Kourtidou C., Tziomalos K. (2023). Epidemiology and Risk Factors for Stroke in Chronic Kidney Disease: A Narrative Review. Biomedicines.

[27] Inserra F., Forcada P., Castellaro A., Castellaro C. (2021). Chronic Kidney Disease and Arterial Stiffness: A Two-Way Path. Front. Med..

[28] Diaz-Ricart M., Torramade-Moix S., Pascual G., Palomo M., Moreno-Castaño A.B., Martinez-Sanchez J., Vera M., Cases A., Escolar G. (2020). Endothelial Damage, Inflammation and Immunity in Chronic Kidney Disease. Toxins.

[29] Rysz J., Mikhailidis D.P., Banach M. (2020). Oxidative Stress and Atherosclerosis in Chronic Kidney Disease. Arch. Med. Sci..

[30] Lo Cicero L., Lentini P., Sessa C., Castellino N., D’ANca A., Torrisi I., Marcantoni C., Castellino P., Santoro D., Zanoli L. (2024). Inflammation and arterial stiffness as drivers of cardiovascular risk in chronic kidney disease. Int. J. Mol. Sci..

[31] London G.M. (2019). Arterial Stiffness in Chronic Kidney Disease and End-Stage Renal Disease. Blood Purif..

[32] Zanoli L., Mikhailidis D.P. (2021). Narrative Review of Carotid disease and the kidney. Ann. Transl. Med..

[33] Siracusa C., Carabetta N., Morano M.B., Manica M., Strangio A., Sabatino J., Leo I., Castagna A., Cianflone E., Torella D. (2024). Understanding Vascular Calcification in Chronic Kidney Disease: Mechanisms and Targets. Int. J. Mol. Sci..

[34] Palamar M., Radulescu I.D.G., Tanasescu M.D., Sircuta A., Bob F. (2025). Vascular calcification in chronic kidney disease and hemodialysis. Medicina.

[35] Ferro C.J., Mark P.B., Kanbay M., Sarafidis P., Heine G.H., Rossignol P., Massy Z.A., Mallamaci F., Valdivielso J.M., Malyszko J. (2018). Lipid management in patients with chronic kidney disease. Nat. Rev. Nephrol..

[36] Charytan D.M., Sabatine M.S., Pedersen T.R., Im K., Park J.-G., Pineda A.L., Wasserman S.M., Deedwania P., Olsson A.G., Sever P.S. (2019). Efficacy and Safety of Evolocumab in Chronic Kidney Disease in the FOURIER Trial. J. Am. Coll. Cardiol..

[37] Di Giovanni G., Nicholls S.J. (2022). Intensive lipid lowering agents and coronary atherosclerosis: Insights from intravascular imaging. Am. J. Prev. Cardiol..

[38] Byambaa E., Roshanravan B., Berglund L. (2025). Lipoprotein(a) and CKD: Insights into New Therapeutic Opportunities. Clin. J. Am. Soc. Nephrol..

[39] O’Donoghue M.L., Rosenson R.S., Gencer B., López J.A.G., Lepor N.E., Baum S.J., Stout E., Gaudet D., Knusel B., Kuder J.F. (2022). Small Interfering RNA to Reduce Lipoprotein(a) in Cardiovascular Disease. N. Engl. J. Med..

[40] Nissen S.E., Ni W., Shen X., Wang Q., Navar A.M., Nicholls S.J., Wolski K., Michael L., Haupt A., Krege J.H. (2025). Lepodisiran—A Long-Duration Small Interfering RNA Targeting Lipoprotein(a). N. Engl. J. Med..

[41] Heerspink H.J.L., Stefánsson B.V., Correa-Rotter R., Chertow G.M., Greene T., Hou F.-F., Mann J.F.E., McMurray J.J.V., Lindberg M., Rossing P. (2020). Dapagliflozin in patients with chronic kidney disease. N. Engl. J. Med..

[42] EMPA-KIDNEY Collaborative Group (2023). Empagliflozin in Patients with Chronic Kidney Disease. N. Engl. J. Med..

[43] Bakris G.L., Agarwal R., Anker S.D., Pitt B., Ruilope L.M., Rossing P., Kolkhof P., Nowack C., Schloemer P., Joseph A. (2020). Effect of Finerenone on Chronic Kidney Disease Outcomes in Type 2 Diabetes. N. Engl. J. Med..

[44] Pitt B., Filippatos G., Agarwal R., Anker S.D., Bakris G.L., Rossing P., Joseph A., Kolkhof P., Nowack C., Schloemer P. (2021). Cardiovascular Events with Finerenone in Kidney Disease and Type 2 Diabetes. N. Engl. J. Med..

[45] Perkovic V., Tuttle K.R., Rossing P., Mahaffey K.W., Mann J.F., Bakris G., Baeres F.M., Idorn T., Bosch-Traberg H., Lausvig N.L. (2024). Effects of Semaglutide on Chronic Kidney Disease in Patients with Type 2 Diabetes. N. Engl. J. Med..

[46] Lincoff A.M., Brown-Frandsen K., Colhoun H.M., Deanfield J., Emerson S.S., Esbjerg S., Hardt-Lindberg S., Hovingh G.K., Kahn S.E., Kushner R.F. (2023). Semaglutide and Cardiovascular Outcomes in Obesity without Diabetes. N. Engl. J. Med..

[47] Adeseun G.A., Xie D., Wang X., Joffe M.M., Iii E.R.M., Townsend R.R., Budoff M., Rosas S.E. (2012). Carotid plaque, carotid intima-media thickness, and coronary calcification equally discriminate prevalent cardiovascular disease in kidney disease. Am. J. Nephrol..

[48] Brady T.M., Schneider M.F., Flynn J.T., Cox C., Samuels J., Saland J., White C.T., Furth S., Warady B.A., Mitsnefes M. (2012). Carotid intima-media thickness in children with CKD: Results from the CKiD study. Clin. J. Am. Soc. Nephrol..

[49] Lopes R., de Morais M.B., Oliveira F.L.C., Brecheret A.P., Abreu A.L.C.S., de Andrade M.C. (2019). Evaluation of carotid intima-media thickness and factors associated with cardiovascular disease in children and adolescents with chronic kidney disease. J. Pediatr..

[50] Bjergfelt S.S., Sørensen I.M.H., Hjortkjær H.Ø., Landler N., Ballegaard E.L.F., Biering-Sørensen T., Kofoed K.F., Lange T., Feldt-Rasmussen B., Sillesen H. (2021). Carotid plaque thickness is increased in chronic kidney disease and associated with carotid and coronary calcification. PLoS ONE.

[51] Doyon A., Hofstetter J., Bayazit A.K., Azukaitis K., Niemirska A., Civilibal M., Kaplan Bulut I., Duzova A., Oguz B., Ranchin B. (2025). Progression of carotid intima-media thickness in children with chronic kidney disease study: Risk factors and impact of blood pressure dynamics. J. Am. Heart Assoc..

